# Optical coherence tomography features of retinal lesions in Chinese patients with endogenous Candida endophthalmitis

**DOI:** 10.1186/s12886-020-01337-9

**Published:** 2020-02-14

**Authors:** Hong Zhuang, Xinyi Ding, Fengjuan Gao, Ting Zhang, Yingqin Ni, Qing Chang, Gezhi Xu

**Affiliations:** 1 Department of Ophthalmology, Eye and ENT Hospital of Fudan University, Shanghai, 200031 China; 2 Key Laboratory of Visual Impairment and Restoration of Shanghai and Key Laboratory of Myopia of State Health Ministry, Fudan University, Shanghai, 200031 China

**Keywords:** Endogenous endophthalmitis, *Candida albicans*, Retinal lesion, Optical coherence tomography

## Abstract

**Background:**

To evaluate the optical coherence tomography (OCT) features of retinal lesions in Chinese patients with endogenous Candida endophthalmitis (ECE).

**Methods:**

We performed a retrospective review of patients diagnosed with ECE at one medical center. The medical records of the patients including predisposing risk factors, treatment and visual acuity were reviewed. And we focused on the analysis of OCT images of retinal lesions before and after treatment.

**Results:**

A total of 16 Chinese patients (22 eyes) were included in this study. The most frequent predisposing risk factors were intravenous use of corticosteroids or antibiotics, lithotripsy for urinary calculi, and diabetes. After treatment, visual acuity was improved in 13 (59.1%) of the 22 eyes, and remained the same in the other 9 (40.9%) eyes. Pre-treatment OCT images obtained at presentation were available for 17 of the 22 eyes. Four types of the OCT manifestations of retinal lesions were identified: type 1 (subretinal macular lesions), type 2 (lesions are located in the inner retinal layer), type 3 (lesions involve the full-thickness retina and accompanied with macular edema), type 4 (sub-inner limiting membrane lesions). Pre-treatment OCT imaging of the 17 eyes revealed five as type 1, four as type 2, six as type 3, and two as type 4. After treatment, OCT images revealed epiretinal membrane and subretinal fibrosis as the most common post-treatment complications of ECE. Epiretinal membrane was detected in 2/4 type 2 lesions, in 4/6 type 3 lesions, and in 1/2 type 4 lesions, while subretinal fibrosis was mainly seen in type 1 lesions (4/5). Among the types, visual prognosis was best in eyes with type 2 lesions.

**Conclusions:**

In this case series, the OCT manifestations of retinal lesions in ECE could be classified into four types. The post-treatment OCT manifestations were different in four types of lesions. We preliminarily found that the OCT morphology of retinal lesions was associated with the visual prognosis of ECE.

## Background

Endogenous endophthalmitis, also called metastatic endophthalmitis, results from hematogenous spread of infectious microbes from distant foci. Endogenous endophthalmitis is a vision-threatening intraocular infection that accounts for approximately 2–10% of all cases of endophthalmitis [1–3], which is reportedly increasing in incidence in East Asian population [4, 5]. The majority of cases of endogenous endophthalmitis are caused by fungal infections [6–8]. Endogenous fungal endophthalmitis is associated with underlying systemic risk factors that include recent major surgery, malignant tumor, systemic antibiotic use, diabetes mellitus, urinary tract infection, and indwelling catheter [9–11]. The majority of these cases are associated with Candida species, especially *Candida albicans* [12–15].

Candida species typically cause inflammation in the choroid, retina, and vitreous. The ophthalmoscopic appearance of endogenous Candida endophthalmitis (ECE) is characterized by one or more white lesions located mainly in the posterior pole of the fundus [16]. Further aggravation of ECE results in vitreous inflammation and disseminated lesions.

Most previous studies have focused on the clinical manifestations of ECE. Several single case studies have reported the manifestations of ECE on optical coherence tomography (OCT) [17–19]. A recent study has analyzed the OCT features of ECE in nine patients from four medical centers in Italy and Australia [20]. Although the incidence of ECE was increased in East Asia, there was still few OCT analysis of ECE in East Asian population.

Therefore, we retrospectively reviewed a case series of Chinese patients with ECE, focusing on the OCT features of ECE before and after treatment. We used OCT for classification and clinical evaluation of retinal lesions caused by Candida infection in patients with ECE.

## Methods

We performed a retrospective review of patients diagnosed with ECE who visited the Eye and ENT Hospital of Fudan University, China, between January 2014 and April 2018. The research followed the tenets of the Declaration of Helsinki and was approved by the Ethics Committee of the Eye and ENT Hospital of Fudan University. Written informed consent was obtained from each patient after explanation of the nature and possible consequences of the study.

ECE was diagnosed according to the patient’s general condition, ocular manifestations, and microbial culture results. Patients with positive *C. albicans* cultures were included. The exclusion criteria were penetrating ocular trauma, recent intraocular surgery, or any other cause of intraocular inflammation. The medical records of the patients were reviewed to obtain the patients’ age, sex, predisposing risk factors, treatment, and visual acuity; and their spectral-domain OCT images were analyzed in detail.

Patients received intraocular therapy and systemic antifungal therapy. Intraocular therapy included intravitreal injection of amphotericin B (5 μg/0.1 ml) and/or vitrectomy. Patients who underwent vitrectomy routinely received an intravitreal injection of amphotericin B at the end of the surgery. All patients received systemic antifungal therapy for at least 2 months. The commonly used daily dose of oral antifungal drugs (fluconazole, itraconazole, or voriconazole) was 400 mg. All patients underwent comprehensive ophthalmic examinations, including slit-lamp biomicroscopy, indirect ophthalmoscopy, measurement of best-corrected visual acuity, B-scan ultrasonography, fundus photography, and spectral-domain OCT.

OCT images were obtained using the Spectralis spectral-domain OCT instrument (Heidelberg Engineering, Heidelberg, Germany). The most obvious retinal lesion in each eye was examined by OCT. After treatment, the eyes were re-scanned using the eye-tracking-based follow-up function in Spectralis OCT. Therefore, the same lesion site was examined, thereby facilitating precise evaluation of the post-treatment changes in the retinal lesions. If OCT images could not be obtained at presentation because of severe vitreous inflammation, the patient underwent OCT examination after vitrectomy.

When comparing the initial visual acuity (before treatment) and the last visual acuity (after treatment), the visual acuity value was converted to logarithm of minimal angle of resolution (LogMAR) unit. According to the literatures [21, 22], visual acuity of counting fingers or worse was converted to LogMAR unit as follows: counting fingers equal to 2.0 LogMAR, hand motion equal to 2.3 LogMAR, light perception equal to 2.5 LogMAR. The paired Wilcoxon rank sum test was performed with Stata 11.0 statistical software (Stata Corporation, College Station, TX, USA). A *P* value of < 0.05 was considered statistically significant.

## Results

### Demographic and clinical characteristics

A total of 16 Chinese patients (22 eyes, 5 males and 11 females) were included in this retrospective observational study. Of these patients, 10 had unilateral ECE and 6 had bilateral ECE. The mean age of the patients was 45.5 ± 14.4 years (range, 23–68 years).

The most frequent predisposing risk factors were intravenous use of corticosteroids or antibiotics (in six patients), lithotripsy of urinary calculi (in four), and diabetes (in three, diabetes combined with nephritis in one). All ECE diagnoses were confirmed by microbial cultures. Ten patients (13 eyes) had vitreous cultures positive for *C. albicans*, and the other six patients (9 eyes) had blood cultures positive for *C. albicans* (two of whom also had a positive midstream urine culture). The positive rate of vitreous fungal culture in vitrectomy samples (78.6%; 11/14) was higher than that in needle biopsy samples (25.0%, 2/8).

Intraocular therapy included intravitreal injection of amphotericin B or vitrectomy; 8 eyes received intravitreal injection of amphotericin B only, and 14 eyes received pars plana vitrectomy. The mean follow-up duration was 7.3 ± 4.1 months (range, 2–18 months). After intraocular therapy and systemic antifungal therapy, the endogenous endophthalmitis caused by Candida infection was effectively controlled. The visual acuity after treatment (mean LogMAR 1.25 ± 0.78) was significantly better than the visual acuity before treatment (mean LogMAR 1.59 ± 0.75) (*P* = 0.0015). The visual acuity was improved in 13 (59.1%) eyes and remained the same in 9 (40.9%) eyes. Eleven (50%) eyes had a visual acuity of 20/200 or better at the last visit. The clinical characteristics of the 22 eyes with ECE are listed in Table 1.

**Table 1:** Clinical characteristics of 22 eyes with ECE

Patient No.	Age/sex	Eye	Predisposing risk factors	C. a culture	Treatment	Initial VA	Last VA
1	41/M	OD	Intravenous corticosteroid	Vitreous (+)	IVI + vitrectomy	20/2000	20/400
2	29/F	OS	Intravenous antibiotics	Vitreous (+)	IVI + vitrectomy	20/2000	20/2000
3	28/M	OS	Endocarditis	blood (+)	IVI + vitrectomy	20/100	20/100
4	40/F	OS	Intravenous corticosteroid	Vitreous (+)	IVI + vitrectomy	HM	HM
5	58/M	OD	Intravenous corticosteroid	Vitreous (+)	IVI	20/200	20/63
		OS		Vitreous (+)	IVI	20/200	20/100
6	23/F	OS	Diabetes	Vitreous (+)	IVI + vitrectomy	HM	20/100
7	67/F	OS	Intravenous corticosteroid	Vitreous (+)	IVI + vitrectomy	CF	CF
8	49/F	OD	nephrolithotripsy	blood (+)	IVI	20/400	20/100
		OS			IVI	20/32	20/25
9	58/F	OD	Diabetes	Vitreous (+)	IVI + vitrectomy	20/2000	20/200
		OS		Vitreous (+)	IVI + vitrectomy	CF	CF
10	37/F	OD	Diabetes; nephritis	blood (+); midstream urine (+)	IVI	20/25	20/20
		OS			IVI + vitrectomy	LP	LP
11	51/F	OD	Intravenous antibiotics	Vitreous (+)	IVI + vitrectomy	CF	20/400
		OS		Vitreous (+)	IVI + vitrectomy	CF	CF
12	53/F	OS	ureterolithotripsy	blood (+)	IVI	20/63	20/50
13	39/M	OD	ureterolithotripsy	Vitreous (+)	IVI + vitrectomy	20/1000	20/1000
14	68/M	OD	nephrolithotripsy	blood (+); midstream urine (+)	IVI	20/100	20/63
		OS			IVI + vitrectomy	CF	20/160
15	59/F	OD	Gastrointestinal surgery	blood (+)	IVI	HM	HM
16	28/F	OD	Abortion	Vitreous (+)	IVI + vitrectomy	HM	CF

*ECE* Endogenous candida endophthalmitis, *F.* Female, *M* Male, *OD* Right eye, *OS* Left eye, *C. a Candida albicans*, *IVI* Intravitreal injection, *VA* Visual acuity, *LP* Light perception, *HM* Hand motion, *CF* Counting fingers

### OCT findings

**Fig. 1:**
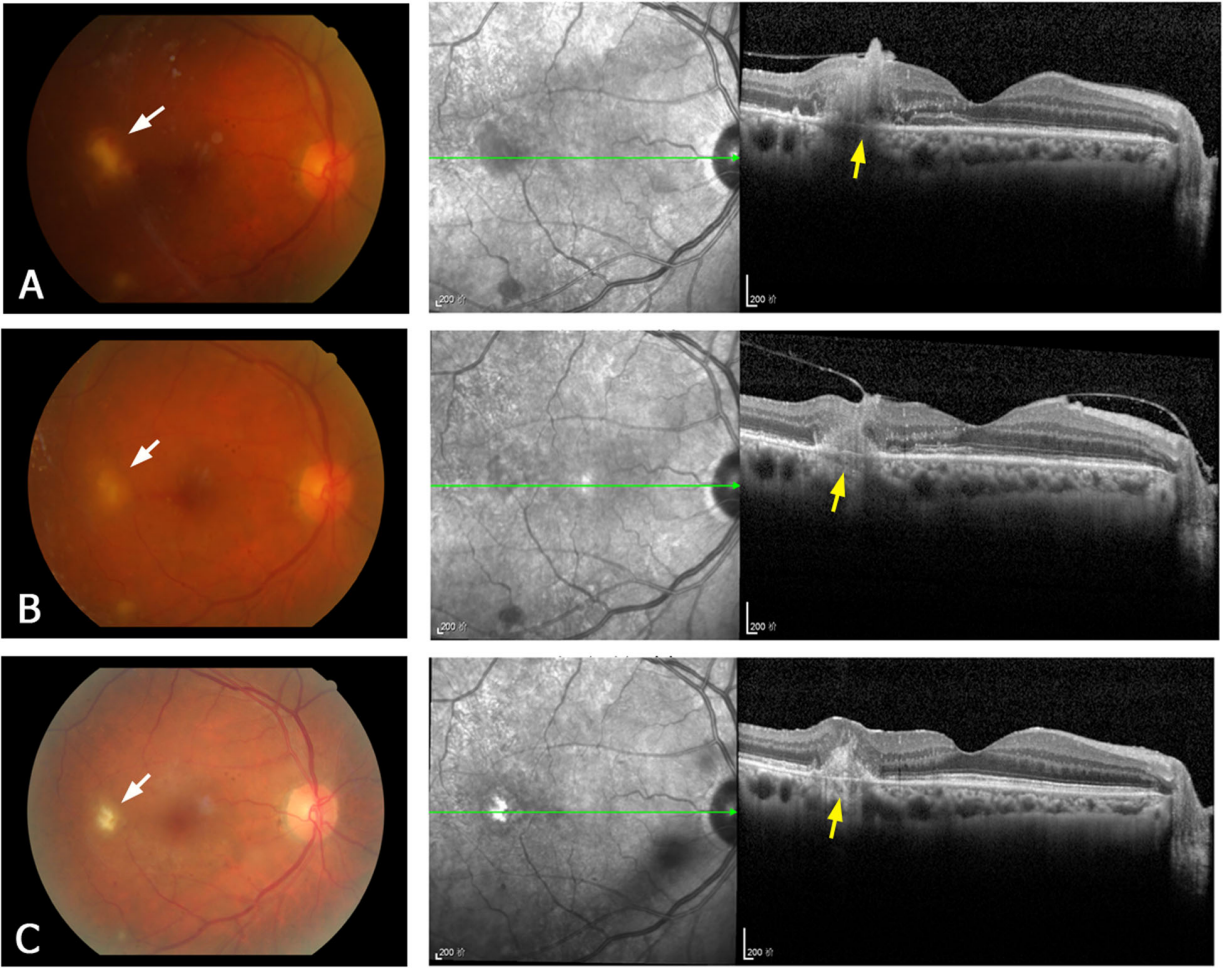
A representative type 1 lesion of ECE. Fundus photographs (left column) and OCT images (right column) of the right eye of patient 5. **a** Before treatment. The fundus photograph shows a yellow–white lesion with a small hemorrhage (white arrow) on the temporal side of the fovea. The OCT image shows a macular subretinal lesion originating at the retinal pigment epithelium/choroid layer (yellow arrow) and penetrating into the neurosensory retina. **b** Two weeks after treatment (intravitreal injection). Shrinkage of the subretinal lesion is apparent on the OCT image. **c** Five months after treatment. The OCT image shows hyperreflective fibrosis in the subretinal lesion. The inner choroid is also hyperreflective

**Fig. 2:**
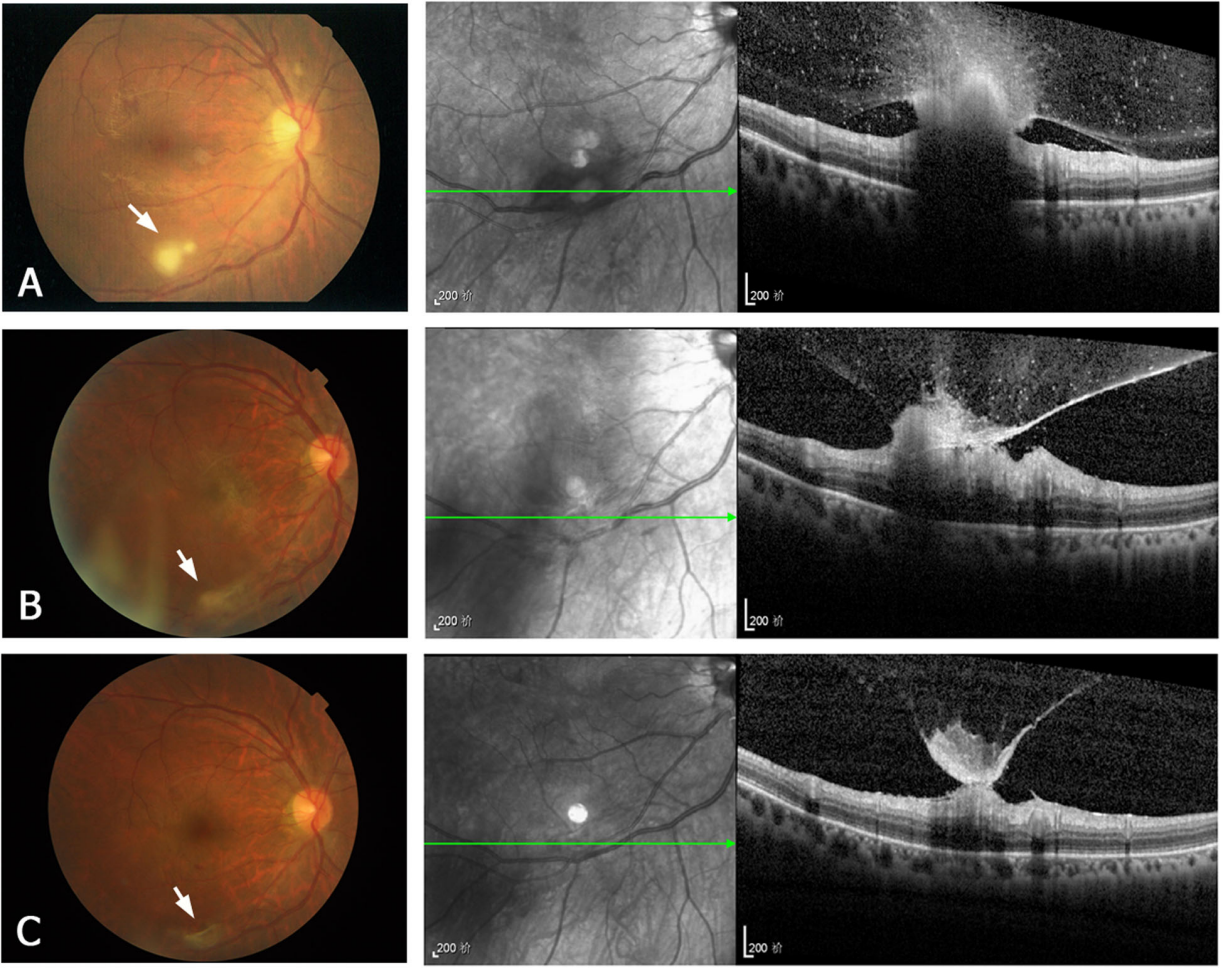
A representative type 2 lesion of ECE. Fundus photographs (left column) and OCT images (right column) of the right eye of patient 10. **a** Before treatment. The fundus photograph shows a white round lesion (approximately one-half disc diameter) near the infratemporal vascular arcade (white arrow). The OCT image shows a lesion in the inner retinal layer, invading the posterior vitreous. No intra- or subretinal fluid is present. **b** One month after treatment (intravitreal injection). The OCT image shows shrinkage of the retinal lesion. **c** Three months after treatment. In the OCT image, regression of the retinal lesion and formation of epiretinal membrane are apparent

**Fig. 3:**
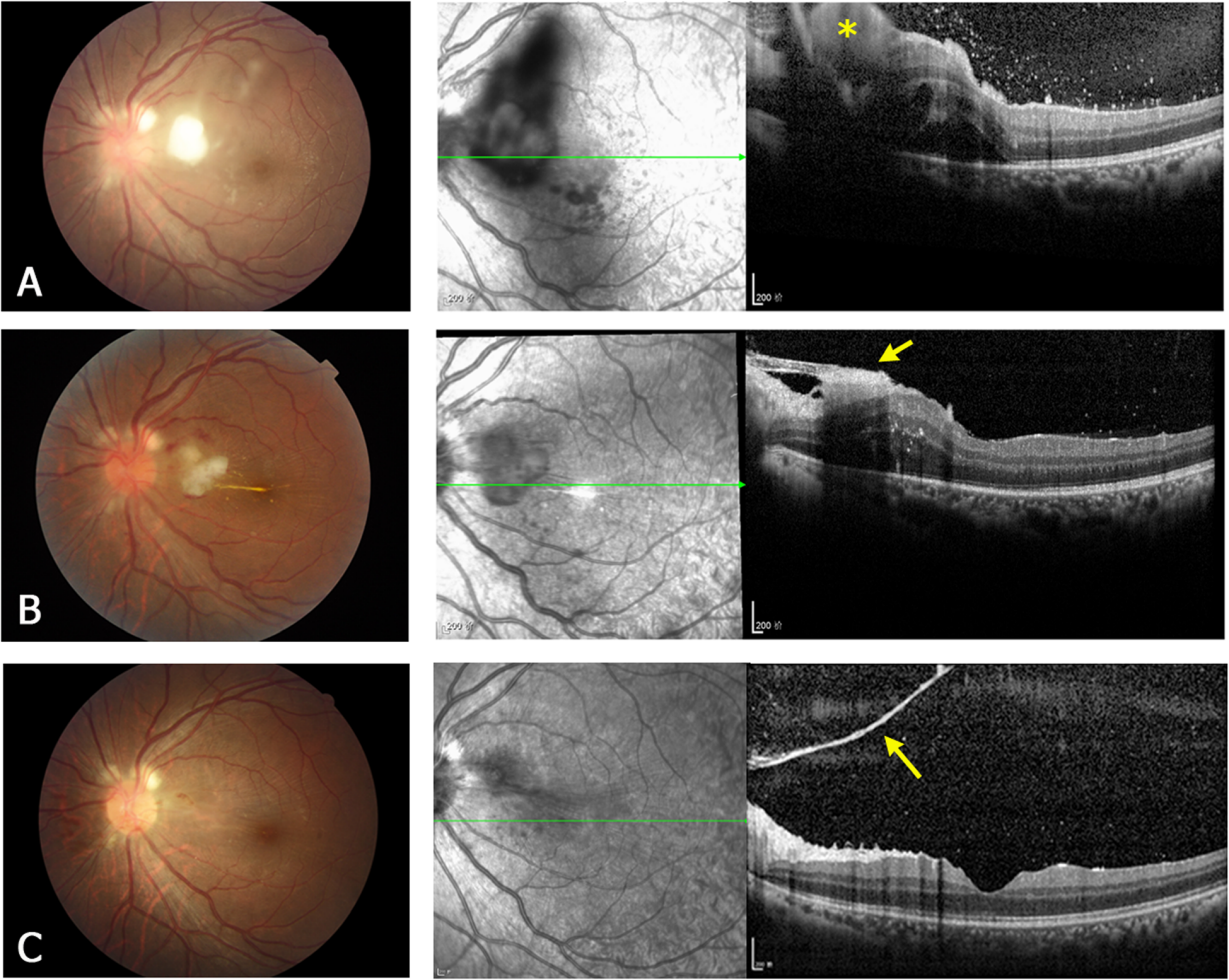
A representative type 3 lesion of ECE. Fundus photographs (left column) and OCT images (right column) of the left eye of patient 3. **a** Before treatment. The fundus photograph shows a white fluffy lesion at the posterior pole. The OCT image shows a highly reflective lesion (yellow asterisk) involving the full-thickness retina and protruding into the vitreous. Macular edema is present with subretinal fluid. The hyperreflective dots in the posterior vitreous are infiltrating inflammatory cells. **b** One week after treatment (vitrectomy). The OCT image shows shrinkage of the retinal lesion, formation of epiretinal membrane (yellow arrow) and reduction of the macular edema. **c** Three months after treatment. The retinal lesion almost resolves, and OCT shows a residual pre-retinal membrane (yellow arrow) close to the optic disc

**Fig. 4:**
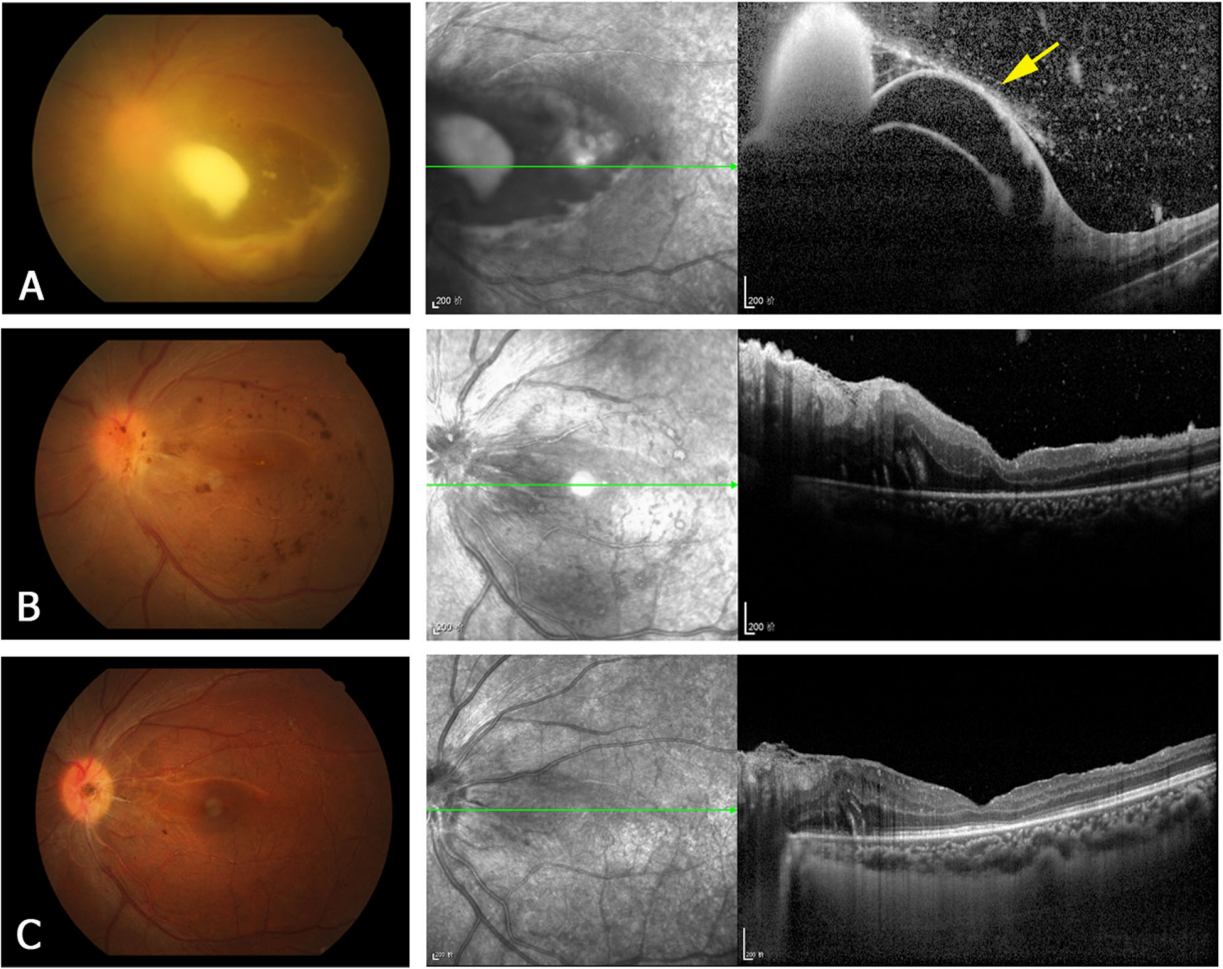
A representative type 4 lesion of ECE. Fundus photographs (left column) and OCT images (right column) of the left eye of patient 6. **a** Before treatment. The fundus photograph shows a large lesion at the posterior pole. The OCT image shows detachment of the ILM (yellow arrow) and the sub-ILM lesion breaking through the ILM into the vitreous. **b** Two weeks after treatment (vitrectomy). In the OCT image, the retinal lesion is no longer apparent, but the nasal retinal edema remains. **c** Three months after treatment. The OCT image shows reduction of the retinal edema and improvement of the macular foveal structure

Pre-treatment OCT was performed at presentation in 17 of the 22 eyes, but not in the other 5 eyes because of severe vitreous inflammation. According to the retinal layer of lesion invasion and the lesion location, four types of the OCT manifestations of the retinal lesions were identified, as follows. **Type 1** are subretinal macular lesions that originate from choroid and penetrate the neurosensory retina (Fig. 1a). **Type 2** lesions are located in the inner retinal layer and protrude into the vitreous, without intra- or subretinal fluid (Fig. 2a). They are always located close to the retinal vascular arcades and are relatively uniform in size, ranging from one-fourth to one disc diameter. **Type 3** lesions involve the full-thickness retina and protrude into the vitreous (Fig. 3a). These lesions always cause macular edema. **Type 4** lesions are located in the sub-inner limiting membrane (ILM) of the macula, and break through the ILM into the vitreous (Fig. 4a). Type 4 lesions exceed 2 disc diameters.

Pre-treatment OCT imaging of the 17 eyes revealed five as type 1, four as type 2, six as type 3, and two as type 4 (Table 2). Table 2 also lists the post-treatment OCT features in the 22 eyes. OCT imaging after treatment revealed regression of the retinal lesion and resolution of the macular edema, and identified epiretinal membrane and subretinal fibrosis as the most common complications of ECE. After treatment, the epiretinal membrane was detected in 2/4 type 2 lesions, in 4/6 type 3 lesions, and in 1/2 type 4 lesions. Among the four types, the subretinal fibrosis mainly existed in type 1 lesions (4/5). The representative OCT follow-up images of each lesion type are shown in Figs. 1, 2, 3 and 4. Table 3 lists the visual prognoses of the different types of retinal lesions. Among the four types, eyes with type 2 lesions had the best baseline and final visual acuities.

**Table 2:** OCT features of 22 eyes with ECE

Case	Eye	OCT pre-treatment	OCT post-treatment
1	OD	Type 3	lesion regressed, ME resolved
2	OS	Type 1	lesion shrunk, fibrosis of lesion
3	OS	Type 3	lesion regressed, ME resolved, ERM
4	OS	NA	lesion regressed, subretinal fibrosis, ERM
5	OD	Type 1	lesion shrunk, fibrosis of lesion
	OS	Type 1	lesion regressed, RPE alteration
6	OS	Type 4	lesion regressed
7	OS	NA	lesion regressed, subretinal fibrosis, ERM
8	OD	Type 3	lesion regressed, subretinal fibrosis, ERM
	OS	Type 2	lesion regressed
9	OD	Type 1	lesion shrunk, fibrosis of lesion
	OS	Type 1	lesion shrunk, fibrosis of lesion
10	OD	Type 2	lesion regressed, ERM
	OS	Type 4	lesion regressed, severe ERM
11	OD	NA	subretinal fibrosis
	OS	NA	subretinal fibrosis, ERM
12	OS	Type 2	lesion regressed, ERM
13	OD	Type 3	lesion regressed, ME reduced
14	OD	Type 2	lesion regressed
	OS	NA	lesion regressed, ERM
15	OD	Type 3	lesion regressed, severe ERM
16	OD	Type 3	lesion regressed, ERM

*OCT* Optical coherence tomography, *ECE* Endogenous candida endophthalmitis, *OD* Right eye, *OS* Left eye, *NA* Non-available (cannot obtain pre-treatment OCT images), *ERM* Epiretinal membrane, *ME* Macular edema, *RPE* Retinal pigment epithelium

**Table 3:** Visual prognosis of different types of retinal lesions in eyes with ECE

	NA	Type 1	Type 2	Type 3	Type 4
Initial VA (> = 20/200)	0/5 (0%)	2/5 (40%)	4/4 (100%)	1/6 (16.7%)	0/2 (0%)
Last VA (> = 20/200)	1/5 (20%)	3/5 (60%)	4/4 (100%)	3/6 (50%)	1/2 (50%)
VA improved	2/5 (40%)	3/5 (60%)	4/4 (100%)	3/6 (50%)	1/2 (50%)

*VA* Visual acuity, *NA* Non-available (cannot obtain pre-treatment OCT images)

## Discussion

ECE is associated with numerous underlying systemic risk factors. In the present patients, the major risk factors for ECE were intravenous use of corticosteroids or antibiotics, lithotripsy of urinary calculi, and diabetes, all of which have been reported previously [9–11]. Other risk factors were endocarditis, gastrointestinal surgery, and abortion [23].

Of the 22 eyes in our study, 14 were treated with vitrectomy. The vitreous opacity and pathogens in the vitreous cavity can be removed by vitrectomy. Vitrectomy is necessary for cases of severe vitreous inflammation and disseminated lesions in the vitreous. If intravitreal injection of amphotericin B cannot control the endophthalmitis, vitrectomy can be performed. It has been reported that the rate of a positive vitreous culture was higher with vitrectomy than with vitreous needle biopsy [8, 24]. Our study confirmed that the positive rate of vitreous fungal culture in vitrectomy samples (78.6%) was higher than that in needle biopsy samples (25.0%).

Several case reports have described the OCT findings of subretinal ECE lesions [17–19]. A recent study [20] analyzed the OCT features of ECE in nine patients from Italy and Australia, and described two OCT patterns of retinal ECE lesions: intraretinal (the lesion is confined to the inner retinal layer or extends into the vitreous) and chorioretinal (the subretinal lesion penetrates through the retina into the vitreous) patterns. In our study involving Chinese patients, four OCT types of retinal ECE lesions were identified. Types 1 (subretinal lesion in the macula) and 2 (lesion in the inner retinal layer) are respectively similar to the chorioretinal and intraretinal patterns described before [20]. Type 3 lesions involve the full thickness of the retina, and cause massive inflammatory cells in the posterior vitreous. Type 3 lesions are always accompanied with macular edema and subretinal fluid, but without obvious subretinal infection lesions. Type 4 lesions are relatively rare but special. The growth of Candida lesions in the retina results in detachment of the ILM. To our knowledge, this is the first report of type 4 lesions in ECE.

There have been very few OCT follow-up studies of ECE after treatment. Even in the abovementioned OCT study [20] of patients with ECE, follow-up images were not available for most patients. In our study, all 22 eyes were followed-up by OCT for at least 2 months at the same medical center, and post-treatment changes in the retinal lesions were evaluated using the eye-tracking-based follow-up function of the OCT instrument. These images revealed the most common complications of ECE after treatment were epiretinal membrane and subretinal fibrosis. We found that the post-treatment OCT manifestations were different in four types of lesions. Epiretinal membrane was detected in type 2 lesions, type 3 lesions, and type 4 lesions, while subretinal fibrosis was mainly seen in type 1 lesions.

As reported previously [25], ECE is associated with a high rate of vision loss. Factors associated with poor visual outcomes include poor baseline visual acuity and centrally located fungal lesions. Our analysis of visual prognosis among the four retinal lesion types revealed that the eyes with type 2 lesions had better baseline visual acuity. After antifungal therapy without vitrectomy, the final visual acuity of all eyes with type 2 lesions was improved. This may be explained by the distance between type 2 lesions and the macular fovea.

Due to the limited number of cases included, this study could not provide a detailed statistical analysis of visual prognosis of different retinal lesions. Further larger sample size study is needed to confirm our OCT classification of retinal lesions and related visual prognosis.

## Conclusion

Compared with the previous reports, this study has broadened our understanding of the OCT features of ECE. In this case series, we preliminarily found that the OCT morphology of retinal lesions was associated with the visual prognosis of ECE. And OCT can be used to monitor the therapeutic effects on ECE.

## Supplementary information

**Additional file 1.** Supplemental Table 1. Symptom duration before treatment in 22 eyes.

## Data Availability

The data used in the current study is available from the corresponding author upon request.

## Abbreviations

C. a: *Candida albicans*
ECE: Endogenous Candida endophthalmitis
ERM: Epiretinal membrane
ILM: Inner limiting membrane
ME: Macular edema
OCT: Optical coherence tomography
RPE: Retinal pigment epithelium
VA: Visual acuity
