# Supplementary material for: Optical coherence tomography features of retinal lesions in Chinese patients with endogenous Candida endophthalmitis
Source: BMC Ophthalmol. 2020 Feb 14;20:52. doi: 10.1186/s12886-020-01337-9 (PMC7020574; doi:10.1186/s12886-020-01337-9)
Supplement: Supplementary file 1 — Additional file 1. Supplemental Table 1. Symptom duration before treatment in 22 eyes. [file 12886_2020_1337_MOESM1_ESM.docx]

Supplemental table 1. Symptom duration before treatment in 22 eyes

| Patient No. | Eye | Symptom duration before treatment (week) | OCT pre-treatment |
| --- | --- | --- | --- |
| 1 | OD | 3 | Type 3 |
| 2 | OS | 2 | Type 1 |
| 3 | OS | 1 | Type 3 |
| 4 | OS | 3 | NA |
| 5 | OD | 3 | Type 1 |
|  | OS | 3 | Type 1 |
| 6 | OS | 2 | Type 4 |
| 7 | OS | 6 | NA |
| 8 | OD | 4 | Type 3 |
|  | OS | 4 | Type 2 |
| 9 | OD | 4 | Type 1 |
|  | OS | 4 | Type 1 |
| 10 | OD | 2 | Type 2 |
|  | OS | 6 | Type 4 |
| 11 | OD | 2 | NA |
|  | OS | 2 | NA |
| 12 | OS | 3 | Type 2 |
| 13 | OD | 4 | Type 3 |
| 14 | OD | 3 | Type 2 |
|  | OS | 4 | NA |
| 15 | OD | 1 | Type 3 |
| 16 | OD | 8 | Type 3 |
